# Cerebrospinal fluid lactate concentration to distinguish bacterial from aseptic meningitis: a systemic review and meta-analysis

**DOI:** 10.1186/cc9395

**Published:** 2010-12-31

**Authors:** Nguyen T Huy, Nguyen TH Thao, Doan TN Diep, Mihoko Kikuchi, Javier Zamora, Kenji Hirayama

**Affiliations:** 1 Department of Immunogenetics, Institute of Tropical Medicine (NEKKEN), Nagasaki University, 1-12-4 Sakamoto, Nagasaki 852-8523, Japan; 2 Department of Pediatrics, University of Medicine and Pharmacy at Ho Chi Minh City, 217 Hong Bang Street, District 5, Ho Chi Minh City, Vietnam; 3 Children's Hospital No.1, 2 Su Van Hanh Street, District 10, Ho Chi Minh City, Vietnam; 4 Center for International Collaborative Research, Nagasaki University, 1-12-4 Sakamoto, Nagasaki 852-8523, Japan; 5 Clinical Biostatistics Unit, Ramón y Cajal Hospital, Carretera de Colmenar km 9.100, 28034 Madrid, Spain; 6 Global COE program, Nagasaki University, 1-12-4 Sakamoto, Nagasaki 852-8523, Japan

## Abstract

**Introduction:**

Making a differential diagnosis between bacterial meningitis and aseptic meningitis is a critical clinical problem. The utility of a cerebrospinal fluid (CSF) lactate assay for this purpose has been debated and is not yet routinely clinically performed. To adequately evaluate this assay, a systematic review and meta-analysis of studies of the CSF lactate concentration as a marker for both bacterial meningitis and aseptic meningitis was performed.

**Methods:**

Electronic searches in PubMed, Scopus, the MEDION database and the Cochrane Library were conducted to identify relevant articles published before March 2009. A manual search of reference lists from selected articles was also conducted. Two reviewers independently selected relevant articles and extracted data on study characteristics, quality and accuracy.

**Results:**

Twenty-five articles were identified that met the eligibility criteria. Diagnostic odds ratios were considerably homogenous (Chi-square *P *= 0.1009, *I*^*2 *^= 27.6%), and the homogeneity was further confirmed by a Galbraith plot and meta-regression analysis using several covariates. The symmetrical summary receiver-operator characteristic curve (SROC), fitted using the Moses-Shapiro-Littenberg method, was positioned near the upper left corner of the SROC curve. The Q value and area under the curve were 0.9451 and 0.9840, respectively, indicating excellent accuracy. The diagnostic accuracy of the CSF lactate concentration was higher than those of other four conventional markers (CSF glucose, CSF/plasma glucose quotient, CSF protein, and CSF total number of leukocytes) using a head to head meta-analysis of the 25 included studies.

**Conclusions:**

To distinguish bacterial meningitis from aseptic meningitis, CSF lactate is a good single indicator and a better marker compared to other conventional markers.

## Introduction

Accurate and rapid diagnosis of acute bacterial meningitis (BM) is essential because disease outcome depends on immediate initiation of appropriate antibiotic therapy [1]. BM should be treated promptly with antibiotics, whereas acute aseptic meningitis (AM) is usually self limiting. However, differentiating BM from AM may be challenging for clinicians because the symptoms and laboratory assays are often similar and overlapping. In addition, classical clinical manifestations of BM in infants and children are usually difficult to recognize because of the absence of signs of meningeal irritation and because of delayed elevation of intracranial pressure. Parameters examined in cerebrospinal fluid (CSF) are less descriptive in children than in adults: in enterovirus meningitis, CSF parameters can be practically identical to those of bacterial meningitis. For example, acute meningitis with predominance of neutrophils in CSF suggests BM; however, herpes simplex-1 infected meningitis presents with > 90% neutrophils in CSF [2]. Furthermore, other assays, such as Gram stain, latex agglutination, and polymerase chain reaction-based assays, lack sensitivity [3-6]. In practice, before definitive CSF bacterial cultures are available, most patients with acute meningitis are treated with broad-spectrum antibiotics targeting BM. In general, this does not seriously harm the AM patient; however, it may enhance the local frequency of antibiotic resistance [7] and cause antibiotic adverse effects, nosocomial infections [8], and high medical costs [9]. Thus, it is not only important to recognize BM patients who promptly need antimicrobial therapy but also AM patients who do not need antibiotics and/or hospital stays.

In recent years, it has been proposed that CSF lactate may be a good marker that can differentiate bacterial meningitis (> 6 mmol/l), from partially treated meningitis (4 to 6 mmol/l) and aseptic meningitis (< 2 mmol/l) [10]. However, other researchers have suggested that CSF lactate offers no additional clinically useful information over conventional CSF markers [11,12]. Other markers, such as C-reactive protein (CRP) [13] and procalcitonin [14], may allow differentiation of patients with bacterial meningitis from those with aseptic meningitis. However, neither of these markers is routinely used in clinical practice [4]. The reported diagnostic accuracy of CSF lactate for the differential diagnosis of BM from AM has varied across studies [11,12]. To adequately evaluate its accuracy, a systematic review and meta-analysis were performed on studies that had investigated the CSF lactate concentration as a differential marker in both BM and AM patients.

## Materials and methods

A protocol was designed before this study was performed as recommended by the Quality of Reporting of Meta-analyses (QUORUM) statement [15] and the PRISMA Statement [16].

### Search strategy and study selection

Four electronic databases, PubMed [17], Scopus [18], MEDION database [19] and the Cochrane Library [20], were searched for suitable studies published before March 2009. The search terms that were used included "meningitis AND (lactate OR lactic)". Only articles written in English that evaluated the CSF lactate/lactic acid concentration for differential diagnosis distinguishing BM from AM were included.

Clinical diagnosis was used as reference standard for BM and AM to avoid misclassification of BM patients as AM. For sub-group analysis, diagnosed BM was defined as a patient with CSF pleocytosis (CSF leukocyte count > 4 cells/μl) and one of the following criteria: (1) positive CSF Gram-stained smear for a bacterial pathogen, (2) positive CSF culture for a bacterial pathogen, (3) positive CSF latex agglutination assay or polymerase chain reaction assay for a bacterial pathogen, or (4) positive blood culture. Diagnosed viral AM was defined as the diagnosis of a patient with pleocytosis in the CSF of ≥ 4 leukocytes/μl combined with the absence of any of the four criteria for BM and with either of the following criteria: a positive polymerase chain reaction assay or a positive culture for viral pathogen or specific antiviral antibodies in CSF and serum [21].

Studies with fewer than 16 participants were excluded in order to limit selection bias (≥ 8 BM patients and ≥ 8 AM patients were required for inclusion) [22]. Furthermore, the following studies were also excluded: (1) animal studies, case reports, replies and reviews; (2) studies in which data could not be extracted; and (3) studies that used lactate as a criteria for diagnosis of AM.

Two independent reviewers (NTH and NTHT) scanned primary titles and abstracts (when available) to select potential full text articles for further scrutiny. When the title and abstract could not be rejected by any reviewer, the full text of the article was obtained and carefully reviewed for inclusion by the two reviewers. Inclusion or exclusion of each study was determined by discussion and consensus between the two reviewers. If multiple reports contained overlapping cases, only the largest report was included. When overlap could not be determined conclusively, the study with the most inclusive information or the latest report was included.

### Data extraction

Two independent investigators (NTH and NTHT) extracted data from the studies chosen for inclusion. Disagreements were resolved by discussion and consensus. Studies with criteria for establishing the diagnosis of BM that relied solely on clinical or laboratory improvement after antibiotic therapy were excluded. In selected studies, the following patients who met the following criteria were also excluded from the BM groups: (1) patients with tuberculous or fungal meningitis, (2) BM patients who received antibiotics before lumbar puncture, (3) post-surgery or traumatic patients, and (4) patients with other central nervous system conditions that could contribute to elevation of CSF lactate (such as recent stroke, seizures, brain hypoxia, and brain trauma). A 2 × 2 diagnostic table was constructed from informative descriptions, lactate values, lactate plots, sensitivity, specificity, likelihood ratios, and receiver-operator characteristic (ROC) curves. Other information for each study, such as author, publication year, age range of patients, assay methods, stabilizer addition versus immediate measurement of lactate, prior antibiotic treatment, tuberculosis, country and city where the study was performed, study design (cross sectional or case control), data collection (prospective or retrospective), assignment of the patient (consecutive or random), and blinded interpretation of lactate measurements and diagnostic results, were also recorded.

### Quality assessment

The quality of included studies was assessed using criteria suggested by Pai *et al.*[23], as it has been observed that these criteria can affect the accuracy of the lactate method. The quality of each study included in the meta-analysis was determined across five metrics: diagnostic criteria, study design, exclusion of patients who received antibiotics before lumbar puncture, exclusion of patients with other disorders, and the method of the lactate assay. Since case-control studies reportedly over-estimate the accuracy result [24], the study design was scored as follows: studies with cross-sectional were assigned one point; those with case-control were assigned zero points. For data collection, prospective studies were identified and assigned two points, retrospective studies were assigned one point, and a study with unknown study design was assigned zero points. In addition, studies that recruited consecutive or random patients were assigned one point, while studies without this kind of information were assigned zero points. Studies excluding chronic diseases or other central nervous disorders patients were assigned one point. Studies that originally excluded data from subjects who received antibacterial therapy prior to lumbar puncture were assigned two points, while studies that included subjects who received antibacterial therapy prior to lumbar puncture and excluded in the present report were assigned one point. Studies that originally excluded data from subjects with TB meningitis were assigned two points, while studies that included these subjects and were excluded by us in this report were assigned one point. For the quality of the method, studies with blinded assessment of the lactate assay with diagnostic results were assigned one point. Since sample processing is another important issue that may affect the accuracy of the assay [25], studies using a stabilizer for lactate sample processing or measuring immediately were assigned one point. Quality was evaluated by discussion and consensus after the independent review of each study by two authors (NTH and NTHT).

### Meta-analysis

Data were analyzed using Meta-Disc (version 1.4) software (Unit of Clinical Biostatistics, Ramón y Cajal Hospital, Madrid, Spain) [26] unless otherwise stated. The software is publicly available [27]. Accuracy measures including sensitivity, specificity, positive likelihood ratio (LR+), negative likelihood ratio (LR-), and diagnostic odds ratio (DOR) were computed. The DOR describes the ratio of the odds of a positive assay in a BM patient compared with a AM patient and was calculated by LR+/LR- (or (sensitivity/(1-specificity))/((1-sensitivity)/specificity)) [28]. A DOR > 1 indicated the assay had discriminative power; a higher DOR indicated more discriminative power.

Heterogeneity of both the sensitivity and specificity across the studies was tested using a *χ*^2 ^test. A *χ*^2 ^*P-*value of < 0.05 was considered heterogeneous. An alternative method to explore the heterogeneity, the *I*^*2 *^index, was also used. The *I*^*2 *^index presents the percentage of total variation across studies that is due to heterogeneity rather than chance [29]. *I*^*2 *^values of > 25%, 50%, or 75% were considered to reflect low, moderate, and high heterogeneity, respectively [29].

Pooling of data was performed if sensitivity and specificity were homogeneous [22]. In the case of heterogeneity, a Spearman rank correlation coefficient (*ρ*) was calculated to measure the extent of correlation between sensitivity and specificity. With the Spearman rank correlation coefficient, if there is a correlation the variation between studies is mainly due to different cut-off values and a summary receiver operating characteristic curve may be modeled [22]. A symmetrical SROC fitting was performed when the DOR was found to be constant. A constant DOR is equivalent to the slope of the fitted regression line at zero (testing whether parameter *b *= 0) [26]. As the natural log of DOR (lnDOR) reflects heterogeneity, heterogeneity was explored by subgroup analysis [22]. This subgroup analysis was performed using a univariate meta-regression analysis in order to evaluate the effect of covariates on diagnostic accuracy (DOR). A Galbraith plot was constructed to further visually assess the heterogeneity of lnDOR and to identify outlier studies [30]. For each study, the ratio of lnDOR/standard error (SE) of the lnDOR (SE(lnDOR)) was plotted against 1/SE(lnDOR), and was represented by a single dot [22]. If the heterogeneity of lnDOR remained between studies, the DerSimonian-Laird random effects model (REM) for fitting SROC was chosen [22], and a *P-*value < 0.05 was considered significant. In addition, the heterogeneity of lnDOR across studies was also examined using multivariable logistic meta-regression analysis with the following covariates as predictor variables: criteria for AM, study design (prospective or retrospective), patient recruitment methods (consecutive or random), assay methods, exclusion criteria, prior antibiotic treatment, tuberculous (TB) meningitis, blinded interpretation of lactate measurement, reliability of the method (stabilizer for lactate sample or immediate measurement), quality assessment score, cut-off points, lactate method, age of participants (child or adult), total number of participants, and effective sample size (ESS) (where ESS = (4*n*_1*_n_2_)/(*n*_1_+*n*_2_)) [31]. The variable with the highest *P-v*alue was excluded from the subsequent round of analysis in the multivariable meta-regression model in a stepwise downward manner. A variable was kept in the model if *P-*value < 0.05. The beta-coefficients and corresponding relative DOR from the meta-regression analysis revealed the effect of each variable on the DOR. If a variable was strongly associated with accuracy, further analysis within sub-groups (with a minimum of three studies per subgroup) was conducted to determine diagnostic accuracy and its SROCs.

To further evaluate the accuracy of the CSF lactate concentration, the Q value and area under the curve (AUC) were calculated from the SROC curves. The Q value is the intersection point of the SROC curve with a diagonal line of the ROC space at which sensitivity equals specificity; a higher Q value indicates higher accuracy. AUC values ≥0.5, 0.75, 0.93, or 0.97 were considered to represent fair, good, very good, or excellent accuracy [32].

### Publication bias

Since publication bias is a concern for meta-analysis, the potential presence of this bias was identified using a funnel plot and Egger test [33]. If publication bias was found, the trim and fill method of Duvall and Tweedie was performed to add studies that appeared to be missing [34,35] using the Comprehensive Meta-analysis software version 2.0 (Biostat Inc. Englewood, NJ, USA) [36]. The pooled DOR and its 95% confidence interval were adjusted after the addition of potential missing studies.

## Results

### Literature search

**Figure 1:**
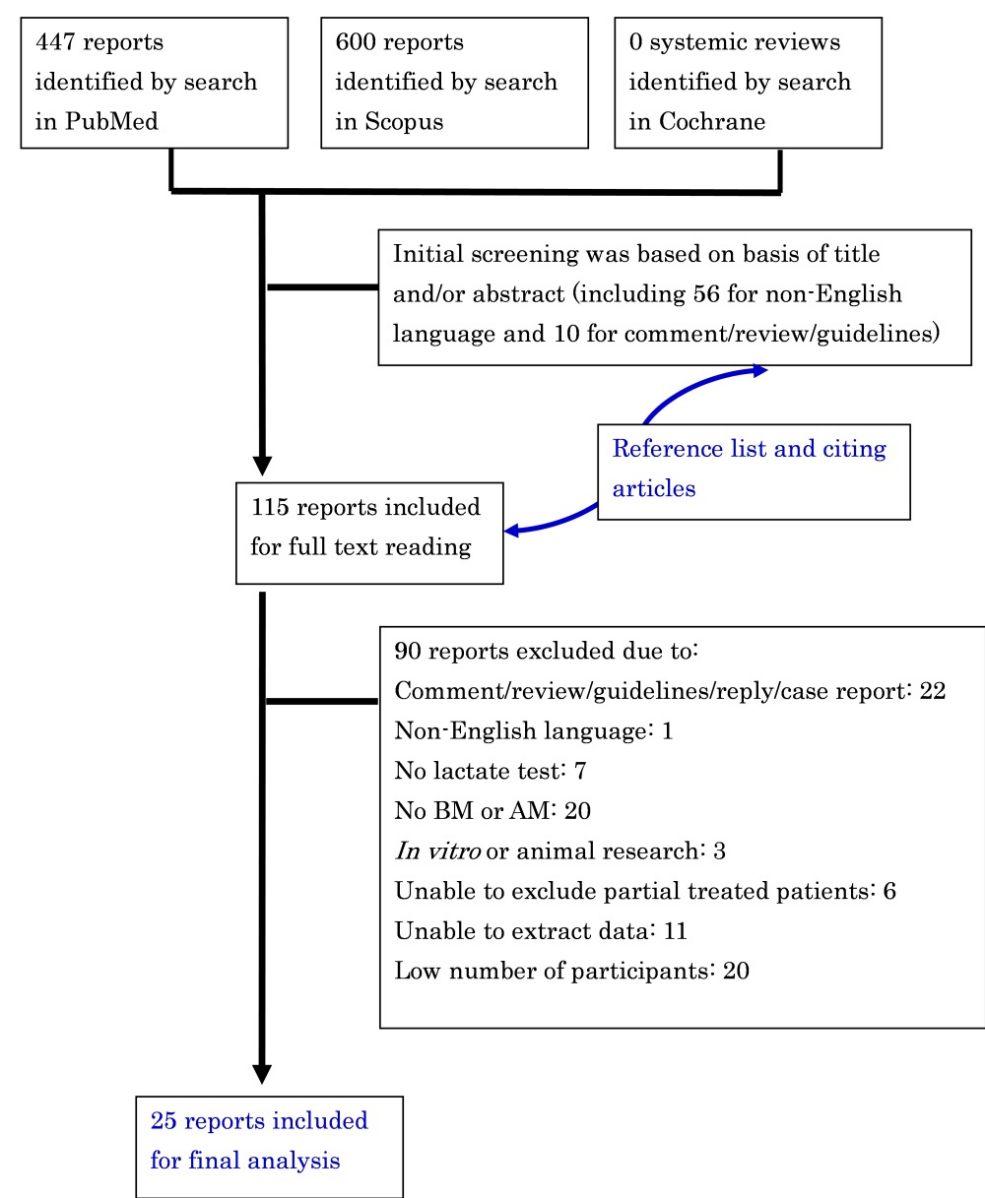
**Flow diagram of the study selection process**.

The literature search initially identified 447 and 600 publications from Pubmed and Scopus, respectively (Figure 1). After an initial screening of the title and/or abstract, 115 articles were included for full text reading. Then additional studies were identified by searching reference lists and articles that cited relevant publications using Scopus databases from full text reviews, review articles, and textbook chapters. These titles and abstracts were reviewed, and the full text was read if necessary. A total of 90 articles were excluded from final analysis due to the following reasons: (1) comment/review/guidelines/reply/case report (*n* = 22), (2) non-English language (*n* = 1), (3) no lactate concentration (*n* = 7), (4) no BM or AM group (*n* = 20), (5) *in vitro *or animal research (*n* = 3), (6) unable to exclude partially treated patients (*n* = 6), (7) unable to extract data (*n* = 11), and (8) low number of participants (*n* = 20). Finally, 25 studies were selected for final analysis [11,12,37-58] with agreement between the two reviewers (κ = 0.898).

The 25 selected publications, which were performed in 16 countries and on five continents, included 783 BM and 909 AM patients. The characteristics of these studies are outlined in Table 1. The average sample size of the included studies was 31 patients (range, 11 to 86) for the BM group and 36 patients (range, 9 to 128) for the AM group. A total of three different methods for lactate measurement (enzymatic: *n* = 19, automatic analyzer: *n* = 2, gas-liquid chromatography *n* = 2) were performed in the 25 included studies. One study used both enzymatic and gas-liquid chromatography methods, with consistent results between the analysis techniques. In all of the 25 included studies, the cut-off value of CSF lactate of < 3.5 mmol/L was applied in 12 studies, while the cut-off value of ≥ 3.5 mmol/L was applied in 12 studies. One study did not indicate the CSF lactate concentration cut-off value.

**Table 1:** Summary of included studies

**Study (ref)**	**Year**	**Country**	**Number of patients**		**Age**	**Lactate method**	**Cut-off (mmol/L)**	**Test results**			
	
			**BM**	**AM**				**TP^a^**	**FP**	**FN**	**TN**
**Abro **[37]	2008	UAE	86	48	Adult	Enz^d^	3.8	85	0	1	48
**Kleine**[59]	2003	Germany	73	128	Adult	Enz	2.61	73	0	0	128
**Schwarz **[58]	2000	Germany	16	14	Adult	NR^c^	2.1	15	8	1	6
**Uduman **[57]	2000	UAE	23	42	Children	Enz	NR	22	3	1	39
**Cameron **[38]	1993	UK	11	9	Children	Enz	4.1	11	0	0	9
**Genton **[39]	1990	Switzerland	19	28	Adult	Auto^e^	4.2	18	0	1	28
**Shaltout **[40]	1989	Kuwait	14	9	Children	Auto	3	13	0	1	9
**Donald **[41]	1986	S. Africa	43	23	Children	Enz	2.85	40	0	3	23
**Nelson **[42]	1986	Sweden	11	28	Children	Enz	2.4	11	3	0	25
**Low **[43]	1986	Singapore	22	54	Children	Enz	2.78	19	8	3	46
**Ruuskanen **[12]	1985	Finland	32	30	Children	Enz	3	30	2	2	28
**Lester **[44]	1985	Denmark	15	15	Child/adult	Enz	4.3	15	0	0	15
**Vanprapar **[45]	1983	Thailand	22	18	Children	Enz	3.89	20	0	2	18
**Mandal **[46]	1983	UK	20	59	Children	Enz	3.9	20	5	0	54
**Pönkä **[47]	1983	Finland	11	27	Child/adult	Enz	3	10	1	1	26
**Briem **[48]	1983	Sweden	45	102	Child/adult	Enz	3.5	45	4	0	98
**Berg **[49]	1982	Sweden	18	121	Child/adult	Enz	3	16	9	2	112
**Eross **[50]	1981	Australia	66	31	Child/adult	Enz	3.9	64	0	2	31
**Knight **[51]	1981	US	68	20	Children	Enz	3.3	68	3	0	17
**Curtis **[52]	1981	UK	13	12	Child/adult	Enz	2.8	13	0	0	12
**Lannigan **[53]	1980	Canada	14	14	Adult	Enz	3.89	13	3	1	11
**Gästrin **[11]	1979	Sweden	38	17	Child/adult	GL^b^	3.5	37	3	1	14
**Lauwers **[54]	1978	Belgium	35	20	NR^c^	GL	3.89	33	0	2	20
**Controni **[55]	1977	US	55	15	Children	Enz&GL	2.78	53	0	2	15
**Bland **[56]	1974	US	13	25	Children	Enz	4.44	12	0	1	25

^a^TP, true-positive; FP, false-positive; FN, false-negative; TN, true-negative; ^b^GL, gas-liquid chromatography; ^c^NR, not reported; ^d^Enz, Enzymatic; ^e^Automatic analyzer.

### Quality of selected studies

In all of the 25 included studies, the lactate assay did not play a role in the final diagnosis of BM or AM. For the study design, 18 studies (72%) were cross-sectional, while seven studies (18%) were case-control studies or not reported (Table 2). Concerning study design, five (21%) collected data prospectively, three (13%) collected data retrospectively, and 16 (69%) did not report the study design. Twelve (50%) studies used either consecutive or random recruitment of participants, while the remaining studies (50%) did not state the method of participant selection. Only one study (4%) described exclusion criteria for participant enrolment, which included the exclusion of patients with chronic diseases or central nervous system disorders. Eleven studies (46%) did not include data from patients who received antibacterial therapy prior to lumbar puncture, seven studies (30%) enrolled subjects who received antibacterial therapy prior to lumbar puncture (these data were excluded in the present report), and six studies (26%) did not mention prior antibacterial therapy. Fourteen studies (58%) originally excluded data from subjects with tuberculous meningitis; eight studies (35%) included these subjects and were excluded in the present study, while no such information could be found in two studies (9%). Concerning the quality of the lactate method, a blinded assessment of the lactate assay with diagnostic results was reported in only three studies (13%), while a stabilizer was used for the lactate sample or an immediate lactate measurement was described in 13 (54%). No study scored the maximal points (11) in the present analysis, while one study received one point. The range of total points was one to eight (Table 2).

**Table 2:** Quality of included studies

Study (ref)	Design^a^	Data collection^b^	Recruit^c^	Exclusion^d^	Prior treatment^e^	TB^f^	Blinded^g^	Reliability^h^	Total score
**Abro **[37]	0	0	0	1	2	2	0	0	5
**Kleine **[59]	1	1	0	0	2	2	0	0	6
**Schwarz **[58]	1	2	1	0	2	0	0	0	6
**Uduman **[57]	1	2	1	0	2	2	0	0	8
**Cameron **[38]	0	0	0	0	2	1	0	1	4
**Genton **[39]	1	1	1	0	1	1	1	1	7
**Shaltout **[40]	1	0	1	0	1	1	1	0	5
**Donald **[41]	0	0	0	0	0	1	0	1	2
**Nelson **[42]	1	1	1	0	1	2	1	1	8
**Low **[43]	0	0	0	0	1	2	0	0	3
**Ruuskanen **[12]	1	0	0	0	0	0	0	0	1
**Lester **[44]	1	2	1	0	1	2	0	1	8
**Vanprapar **[45]	1	0	0	0	0	0	0	0	1
**Mandal **[46]	1	0	1	0	1	2	0	1	6
**Pönkä **[47]	1	0	1	0	0	2	0	0	4
**Briem **[48]	0	2	0	0	2	1	0	0	5
**Berg **[49]	1	2	1	0	2	1	0	1	8
**Eross **[50]	1	2	0	0	2	2	0	1	8
**Knight **[51]	0	0	0	0	0	2	0	1	3
**Curtis **[52]	1	0	1	0	2	1	0	1	6
**Lannigan **[53]	1	0	1	0	2	2	0	0	6
**Gästrin **[11]	1	0	0	0	0	2	0	1	4
**Lauwers **[54]	1	0	1	0	2	1	0	0	5
**Controni **[55]	1	0	1	0	1	1	0	1	5
**Bland **[56]	0	0	0	0	2	2	0	1	5

^a^Study design (cross-sectional or case-control); ^b^Data collection (prospective or retrospective); ^c^recruitment of the patient (consecutive or random); ^d^exclusion criteria; ^e^prior antibiotic treatment; ^f^tuberculous meningitis; ^g^blinded interpretation of lactate measurement; ^h^reliability of the method (stabilizer for lactate sample or immediate measurement).

### Meta-analysis

**Figure 2:**
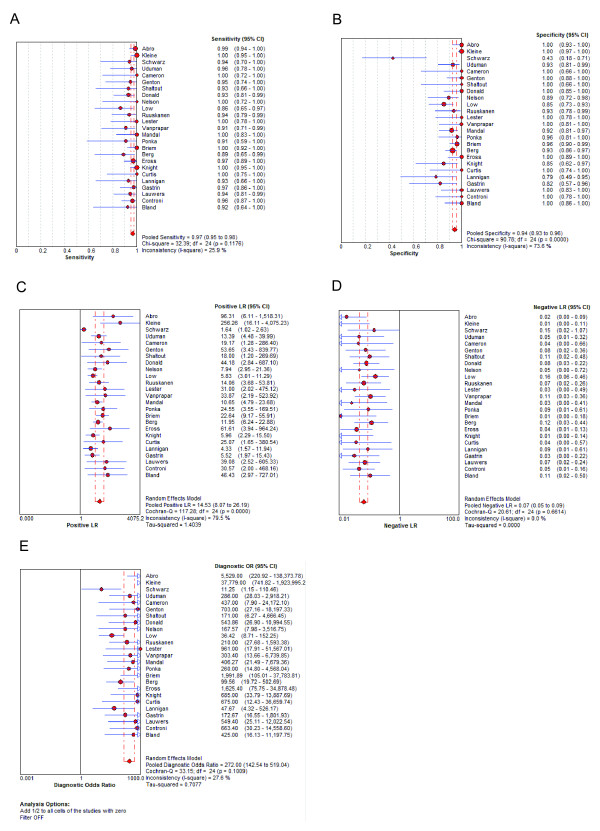
**Diagnostic accuracy of the CSF lactate concentration for differential diagnosis of BM from AM**. Forest plot showing sensitivity, specificity, LR+, LR-, and DOR with 95% confidence intervals (95% CI) for the lactate concentration for differential diagnosis of BM from AM. The size of the circle represents the study size.

The sensitivity of included studies ranged from 0.86 to 1.00 (mean, 0.96; 95% confidence interval (CI), 0.95 to 0.98) (Figure 2), while the specificity varied widely from 0.43 to 1.00 (mean, 0.94; 95% CI, 0.93 to 0.96). The mean of LR+ was calculated at 14.53 (95% CI, 8.07 to 26.19), LR- at 0.07 (95% CI, 0.05 to 0.09) and the mean DOR was 270.0 (95% CI, 142.54 to 519.04).

**Figure 3:**
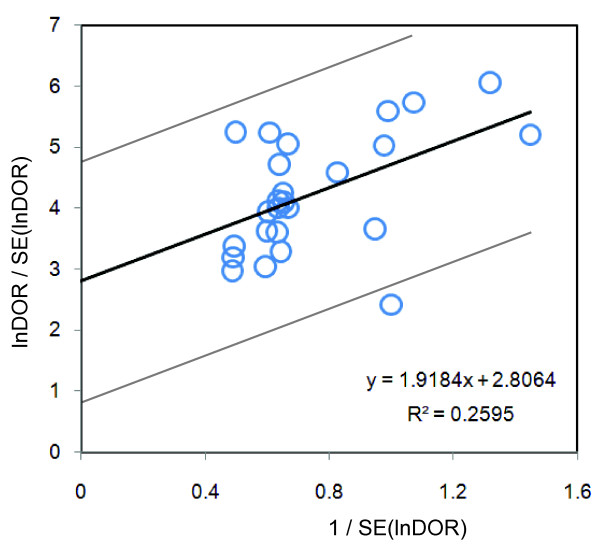
**Galbraith plot of the CSF lactate concentration for differential diagnosis of BM from AM**. The horizontal axis represents lnDOR/SE(lnDOR), while the vertical axis represents 1/SE(lnDOR). The regression runs through the origin interval (central solid line). The 95% confidence interval is between the two outer parallel lines at two units above and below the regression line.

Heterogeneity was present among the studies with regard to specificity (*χ*^2 ^*P *= 0.000, *I*^*2 *^= 73.6%), and to LR+ (*χ*^2 ^*P *= 0.000, *I*^*2 *^= 79.5%). Therefore, pooling of data was not performed [22]. Because of the significant heterogeneity of these data, the Spearman rank correlation coefficient (*ρ*) was calculated to measure the extent of correlation between sensitivity and specificity. The present results indicated a poor correlation between sensitivity and specificity, with a Spearman *P *= -0.043, suggesting that variation between studies was not mainly due to different cut-off values [22]. In contrast, homogeneity was present among the studies with regard to sensitivity (*χ*^2 ^*P *= 0.12, *I*^*2 *^= 25.9%), LR- (*χ*^2 ^*P *= 0.66, *I*^*2 *^= 0.0%), and for DOR (*χ*^2 ^*P *= 0.1009, *I*^*2 *^= 27.6%). A Galbraith plot was created to graphically assess the homogenous nature of the lnDOR, and to identify potential outlier studies (Figure 3). On the Galbraith plot, 24 studies were inside the 95% bounds (the zones of two outer parallel lines drawn at two units over and below the regression) from the standardized mean lnDOR, while only one study was the outlier [58]. However, the DOR was just slightly increased from 270.0 to 292.71 after removing the outlier study. further confirming the relatively homogenous nature of the lnDOR [22]. The homogenous nature of the lnDOR across studies was also examined using meta-regression analysis with the following covariates as predictor variables: data collection, study design (prospective or retrospective), recruitment of the patient (consecutive or random), assay methods, exclusion criteria, prior antibiotic treatment, tuberculous meningitis, blinded interpretation of lactate measurement, reliability of the method (lactate sample stabilizer or immediate measurement), quality assessment score, cut-off points, lactate method, age of participants (children/adult), total number of participants, and effective sample size (ESS). The present results revealed an independent association of the lnDOR with tested covariates (Data not shown). These data suggest that the lnDOR of the included studies is homogenous, and thus a SROC can be fitted based on the pairs of sensitivity and specificity of the individual studies [22].

**Figure 4:**
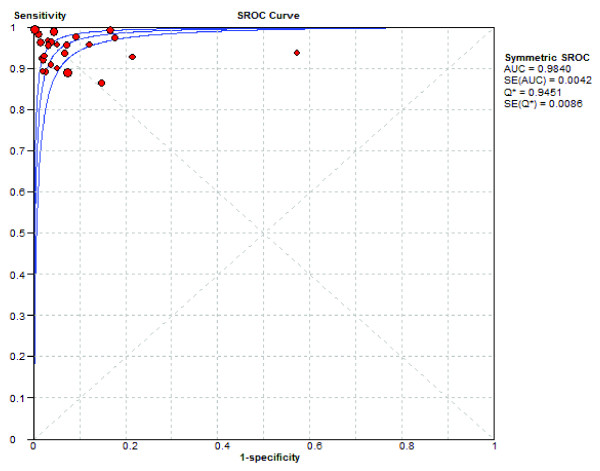
**SROC curve of the CSF lactate concentration for differential diagnosis of BM from AM**. Each circle indicates an individual study in the meta-analysis (*n* = 25). The curve is the regression that summarizes the overall diagnostic accuracy. SE(AUC), standard error of AUC; SE(Q*), standard error of the Q* value. The size of the circle represents the study size.

The slope of the fitted regression line of the Moses-Shapiro-Littenberg model was zero (testing whether parameter *b *= 0, *P *= 0.84), indicating a constant DOR. Therefore, a symmetrical SROC fitting was performed (Figure 4). The present results showed that the SROC curve was positioned near the upper left corner of the SROC curve, with the Q value and AUC at 0.9451 and 0.9840, respectively, indicating excellent accuracy.

### Sub meta-analysis of lactate as a differential marker for diagnosed BM from AM

Meta-analysis was further performed to assess the diagnostic accuracy of lactate between diagnosed BM and AM. Nineteen studies [11,12,38,39,41-43,46-56,59] that analyzed only diagnosed BM and five other studies [37,40,44,45,57] that included diagnosed BM as well as clinical BM that could be extracted separately were included in the subgroup analysis. The specificity and LR+ were heterogeneous among the studies, but sensitivity, LR-, and DOR were significantly homogenous (data not shown). Symmetrical SROC fitting was also performed for these five studies due to a constant DOR (testing whether parameter *b *= 0, *P *= 0.4452). The result showed a SROC curve with the Q value and AUC at 0.9426 and 0.9828, respectively, indicating excellent accuracy, and was consistent with the 25 included studies (data not shown).

### Sub meta-analysis of lactate as a differential marker for diagnosed BM from diagnosed viral AM

Meta-analysis was further performed to assess the diagnostic accuracy of lactate between diagnosed BM and diagnosed viral AM. One study that recruited only diagnosed viral AM and four other studies that included diagnosed viral AM as well as clinical AM that could be extracted separately were included in the subgroup analysis. The specificity was still heterogeneous among the studies (*χ*^2 ^*P *= 0.14, *I*^*2 *^= 42.1%) of diagnostic accuracy, but sensitivity, LR+, LR-, and DOR were significantly homogenous (data not shown). Symmetrical SROC fitting was also performed for these five studies due to a constant DOR (testing whether parameter *b *= 0, *P *= 0.9145). The result revealed a SROC curve with the Q value and AUC at 0.9563 and 0.9891, respectively, suggesting excellent accuracy, and was consistent with above results (data not shown).

### Head-to-head comparison of CSF lactate level versus conventional markers

In order to compare the diagnostic accuracy of the CSF lactate concentration and other conventional markers for diagnosis of BM, data were extracted from the 25 selected articles only if the study had on the same set of specimens a parallel analysis of CSF lactate and a conventional marker. Since conventional markers were used as the diagnostic criteria of BM, only BM patients with confirmed diagnosis were extracted in this analysis. The extracted data are shown in Table 3, which includes the DOR values for CSF lactate, CSF glucose, CSF/plasma glucose quotient, CSF protein, CSF total number leukocytes, CSF percentages of granulocytes, and CSF number of granulocytes.

**Table 3:** Head-to-head comparison of CSF lactate concentration and other conventional markers

Study (ref)	Conventional markers (location)	Conventional markers assay results					Lactate assay results				
		**TP**	**FP**	**FN**	**TN**	**DOR**	**TP**	**FP**	**FN**	**TN**	**DOR**
**Shaltout^a,b,c ^**[40]	Glucose (CSF)	10	1	6	41	68.3	14	1	2	41	287.0
**Donald^a,c ^**[41]		33	2	15	67	73.7	45	3	3	69	345.0
**Pönkä **[47]		5	0	5	27	55.0	10	1	1	26	260.0
**Briem^a,c ^**[48]		30	0	23	193	502.3	47	4	0	167	3,536.1
**Lannigan **[53]		11	4	2	10	13.8	13	3	1	11	47.7
**Genton^a,b ^**[39]	Glucose quotient (CSF/plasma)	21	0	2	27	473.0	24	0	1	28	931.0
**Nelson^b ^**[42]		10	0	7	26	74.2	18	3	0	25	269.6
**Briem^a,c ^**[48]		40	1	13	191	587.7	47	4	0	167	3,536.1
**Berg^a ^**[49]		16	10	2	78	62.4	16	9	2	112	99.6
**Genton^a,b ^**[39]	Protein concentration (CSF)	18	0	3	25	269.6	24	0	1	28	931.0
**Shaltout^a,b,c ^**[40]		13	0	3	42	327.9	14	1	2	41	287.0
**Donald^a,c ^**[41]		39	1	9	68	294.7	45	3	3	69	345.0
**Vanprapar **[45]		8	0	3	12	60.7	12	0	1	18	308.3
**Pönkä **[47]		10	11	1	16	14.5	10	1	1	26	260.0
**Briem^a,c ^**[48]		41	7	9	184	119.8	47	4	0	167	3,536.1
**Berg^a ^**[49]		11	14	4	88	17.3	16	9	2	112	99.6
**Genton^a,b ^**[39]	Leukocytes (CSF total number)	16	0	8	26	102.9	24	0	1	28	931.0
**Shaltout^a,b,c ^**[40]		10	2	6	39	32.5	14	1	2	41	287.0
**Nelson^b ^**[42]		17	1	1	26	442.0	18	3	0	25	269.6
**Pönkä **[47]		7	2	4	25	21.9	10	1	1	26	260.0
**Lannigan **[53]		12	2	2	12	36.0	13	3	1	11	47.7
**Genton^a,b ^**[39]	Granulocytes (CSF %)	20	7	4	19	13.6	24	0	1	28	931.0
**Pönkä **[47]	Neutrophils (CSF number)	7	6	4	21	6.1	10	1	1	26	260.0

^a^Included TB meningitis; ^b^included prior treated patients; ^c ^included normal patients in the AM group. TP, true-positive; FP, false-positive; FN, false-negative; TN, true-negative; DOR, diagnostic odds ratio.

**Figure 5:**
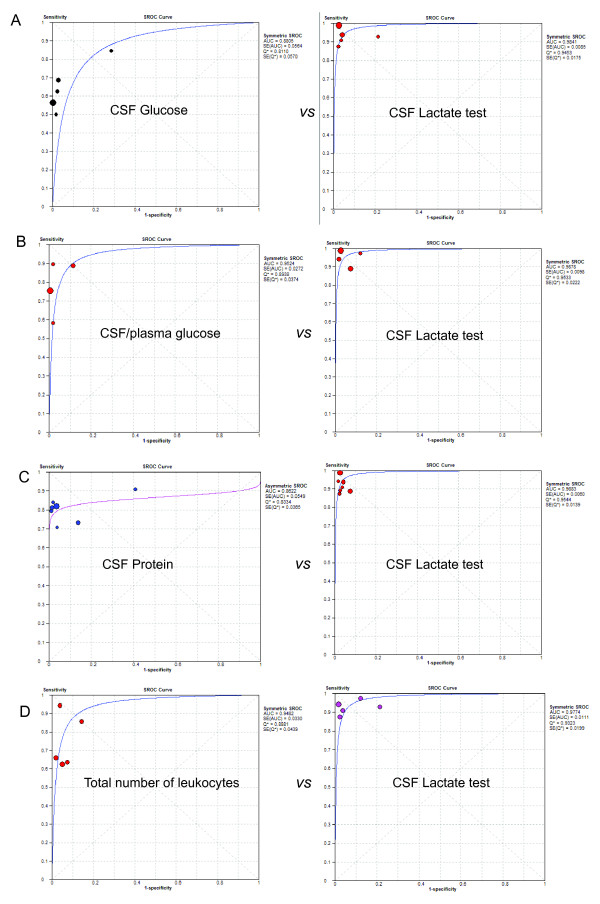
**SROC curve of the head to head comparison of the CSF lactate concentration and other conventional markers**. Each circle indicates an individual study in the meta-analysis. The curve is the regression that summarizes the overall diagnostic accuracy. SE(AUC), standard error of AUC; SE(Q*), standard error of Q* value. The size of the circle represents the study size.

In the present study, for diagnosis of BM, five studies performed head to head comparisons of CSF lactate versus CSF glucose, four versus the CSF/plasma glucose quotient, seven versus CSF protein, five versus CSF total number of leukocytes, one versus percentages of granulocytes, and one versus CSF number of granulocytes. However, TB meningitis patients and partially treated BM patients could not be excluded from the conventional markers assays. Therefore, in a secondary meta-analysis these patients were included in the BM group. Higher DOR values were observed with the CSF lactate level than with the conventional markers in all studies except for one study for the CSF protein assay [40] and one study for total number of leukocytes [42]. Since DOR values of the CSF lactate concentration, CSF glucose level, CSF/plasma glucose quotient, and CSF total number of leukocytes were found to be constant (data not shown), symmetrical SROC fitting by a random effects model was performed for these assays. On the other hand, asymmetrical SROC fitting by a random effects model was computed for the CSF protein assay because the slope of the fitted regression line of the Moses-Shapiro-Littenberg model was not zero (data not shown). Following SROC analysis for all four subgroups of the CSF lactate concentration (Figure 5), the overall AUC was 0.977 to 0.988, which was consistent with the primary analysis of the 25 included studies. In addition, the AUC values were found to be lower for the four conventional markers (0.881, 0.952, 0.862, and 0.948 for CSF glucose, CSF/plasma glucose quotient, CSF protein, and CSF total number of leukocytes, respectively), suggesting a lower accuracy compared to the CSF lactate test.

### Assessment of publication bias

**Figure 6:**
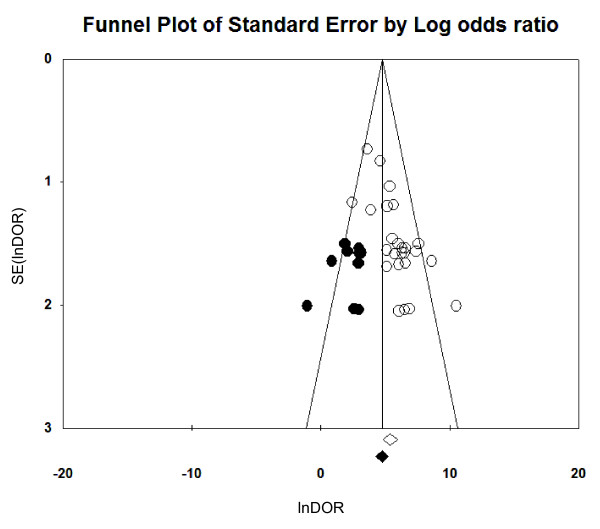
**Funnel plots for evaluation of publication bias in the 25 included studies**. The funnel graph plots the standard error of the lnDOR (SE(lnDOR)) against the log of the diagnostic odds ratio (lnDOR). Each empty circle represents one observed study in the meta-analysis, while an empty diamond indicates the original pooled lnDOR and its 95% confidence interval. The Trim and Fill method was used to find unpublished studies (filled circles) and compute the true vertical line center of the funnel and the adjusted pooled lnDOR (filled diamond) after adding in missing studies (depicted with black dots).

The relatively asymmetric funnel plot (Figure 6) and the Egger intercept (2.95, two-tailed *P *= 0.00004) suggested the presence of a publication bias. Using the trim and fill method of Duvall and Tweedie, 11 missing studies were required in the left side of the funnel plot in order to make the plot symmetric. However, the pooled lnDOR dropped just slightly from 5.60 (95% CI, 4.95 to .25) to 4.84 (95% CI, 4.16 to 5.53) after addition of these missing studies.

## Discussion

The present meta-analysis revealed that the AUC of CSF lactate concentration was 0.9840 (Figure 4), indicating an excellent level of overall accuracy. The overall performance was highest for the CSF lactate concentration compared to the performances of the four conventional markers (CSF glucose, CSF/plasma glucose quotient, CSF protein, and CSF total number of leukocytes) based on head-to-head meta-analytic SROC curves and their AUC (Figure 5), which was in good agreement with previous literature [4,59]. CSF lactate is less useful if it has a low concentration, but the assay is supportive if it is positive, especially if the diagnosis was otherwise not conclusive. In such cases, increased CSF lactate should be considered a sign of BM. Because of the lactate assay, several BM patients with elevated CSF lactate and minimal CSF abnormalities have been treated with antibiotics prior to culture test results [11,47,55]. Moreover, an increased CSF lactate level has been also proposed as a good indicator of CSF infection in intra-ventricular hemorrhagic patients with an external ventricular drain [60,61]. However, clinicians should be aware that CSF lactate is also increased in several central nervous system diseases such stroke (2 to 8 mmol/l) [62,63], convulsion (2 to 4 mmol/l) [64], cerebral trauma (2 to 9 mmol/l) [52], hypoglycemic coma (2 to 6 mmol/l) [65].

The measurement of CSF lactate concentration is a simple, rapid, inexpensive assay, takes just 15 minutes, and can be performed at the bedside. In addition, the CSF lactate concentration is useful during the course of treatment, because a rapid CSF lactate decrease is indicative of good prognosis [39]. Since the CSF lactate concentration is not specific for BM, the results of this assay should be interpreted in parallel with clinical findings and the results of conventional assays including CSF concentrations of protein, cells, glucose, and a microbiological examination of CSF. The cut-off value for CSF lactate concentration ranges from 2.1 to 4.44 mmol/L, suggesting a variance between instrument, hospital labs, and the method. Therefore, every center should set its own cut-off value for CSF lactate concentration. Another disadvantage of CSF lactate is that it is not useful in the choice of antibiotic selection, which must be based on the results of microscopic examination of a smear or culture for bacteria, as well as the other clinical data.

The mechanism of the increased concentration of lactate in the CSF of patients with BM is not clear, but it has been linked with anaerobic glycolysis of brain tissue due to a decrease cerebral blood flow and oxygen uptake [66,67]. Additionally, the concentration of CSF lactate is independent of serum lactate, probably due to its ionized state that crosses the blood-CSF barrier at a very slow rate [68], suggesting another advantage over CSF glucose assay [38].

The present systemic review has several strengths. First, the criteria and protocol were defined, the protocol was followed, and a search of several databases and sources was performed to identify potential studies. The quality of included studies was assessed by using several criteria that could affect diagnostic accuracy. These steps were carried out by two independent researchers. Heterogeneity was explored in accordance with published guidelines. Then, the summary ROC curve was computed and Q values and AUC were calculated in order to evaluate the diagnostic accuracy of CSF lactate marker. Potential effects of several covariates on the diagnostic accuracy were assessed, but none were found.

Because publication bias can affect the accuracy of diagnostic assays, potential publication bias was assessed using funnel plots. The results showed a skewed funnel shape, suggesting a potential publication bias in the literature (Figure 6). However, it was noted that the three largest studies [37,48,50] had higher DORs compared to smaller studies, and they had similar almost perfect accuracy [38,44,56]. This discrepancy could be explained by the calculation method of adding 0.5 to cells with zero, suggesting a weakness of the funnel plot when the assay investigated has excellent accuracy. Another main concern is the lack of some additional databases that were used for searching, that is, we did not access EMBASE, which could have added more relevant studies. we did search Scopus, which is reportedly 91.6% overlapped with EMBASE [69]. Therefore, we think that we have not missed many studies large enough to change the overall impression of our results.

In addition, non-English language studies were also excluded; the non-English language reports represented approximately 10% of all initial articles. We excluded non-English articles in meta-analyses due to limited resource and potential error in the translation and interpretation in several languages including Chinese, Croatian, Dutch, French, German, Hebrew, Italian, Korean, Norwegian, Polish, Portuguese, Romanian, Russian, Serbian, Spanish, and Turkish. The odds ratio in meta-analyses from non-English articles is reportedly 0.8 (95% CI, 0.7 to 1.0) times lower than that from English-written publications [70], therefore, it is unlikely that the inclusion of these non-English articles would have altered our main conclusions substantially.

In addition, studies that reported non-significant results are less likely to be accepted for publication. All of these potentially missing data could result in a significant publication bias. However, the trim and fill method of Duvall and Tweedie was used to overcome this bias, and it was found that it was unlikely to distort the overall diagnostic performance of the lactate concentration (Figure 6).

## Conclusions

The present meta-analysis study indicated that for discrimination of BM from AM, the CSF lactate concentration is a good single indicator and a better marker compared to other conventional markers including CSF glucose, CSF/plasma glucose quotient, CSF protein, and CSF total number of leukocytes. Cost-effectiveness studies should be performed to investigate the economic impact of using this technique as a routine assay in hospital to distinguish BM from AM.

## Key messages

• The diagnostic accuracy of cerebrospinal fluid (CSF) lactate assay for differential diagnosis between bacterial meningitis and aseptic meningitis was excellent with Q value of 0.9451 and area under the curve of 0.9840.

• CSF lactate was a better marker for distinguishing bacterial meningitis from aseptic meningitis compared to other conventional markers including CSF glucose, CSF/plasma glucose quotient, CSF protein, and CSF total number of leukocytes

## Abbreviations

AM: aseptic meningitis; AUC: area under the curve; BM: bacterial meningitis; CI: confidence interval; CRP: C-reactive protein; CSF: cerebrospinal fluids; DOR: Diagnostic odd ratio; ESS: effective sample size; lnDOR: natural log of DOR, LR+, positive likelihood ratio; LR-: negative likelihood ratio, ROC, receiver-operator characteristic; SE: standard error; SROC: summary ROC curve; TB: tuberculosis.

## Competing interests

The authors declare that they have no competing interests.

## Authors' contributions

NTH designed the research, collected, analyzed and interpreted the data, and drafted and revised the manuscript. NTHT carried out the collection, analysis and interpretation of the data. DTND and MK contributed to the conception of the study and approved the final version of the manuscript. JZ helped to design the study, performed the statistical analysis and drafted the manuscript. KH participated in the design of the study and drafted and revised the manuscript. All authors read and approved the final manuscript.
